# Intraoperative subcutaneous culture as a predictor of surgical site infection in open gynecological surgery

**DOI:** 10.1371/journal.pone.0244551

**Published:** 2021-01-12

**Authors:** Ricardo Sainz de la Cuesta, Rosa Mohedano, Sylvia Sainz de la Cuesta, Belen Guzman, Alicia Serrera, Silvia Paulos, Margarita Rubio

**Affiliations:** 1 Department of Obstetrics and Gynecology, Hospital Universitario Quironsalud Madrid, Madrid, Spain; 2 Department of Medicine, Faculty of Biomedical and Health Sciences, Universidad Europea de Madrid, Madrid, Spain; 3 Department of Microbiology, Hospital Universitario Quironsalud Madrid, Madrid, Spain; University of Insubria, ITALY

## Abstract

**Purpose:**

To analyze the relationship between intraoperative cultures and the development of surgical site infection (SSI) in women undergoing laparotomy for gynecological surgery.

**Methods:**

Prospective observational cohort study. Over a six-year period, women who underwent elective laparotomy at our hospital were included. Patients’ demographics, underlying co-morbidities, surgical variables, type and etiology of postoperative surgical site infections were collected. Skin and subcutaneous samples were taken just prior to skin closure and processed for microbiological analysis. Univariate and multivariate analyses (logistic regression model) were conducted to explore the association of the studied variables with SSIs.

**Results:**

284 patients were included in our study, of which 20 (7%) developed surgical site infection, including 11 (55%) superficial and nine (45%) organ-space. At univariate analysis, length of surgery, colon resection, transfusion and positive intraoperative culture were associated with surgical site infection occurrence. Skin and subcutaneous cultures were positive in 25 (8.8%) and 20 (7%) patients, respectively. SSI occurred in 35% of women with positive subcutaneous culture and in 20% of those with positive skin cultures. Using multivariate analysis, the only independent factor associated with surgical site infection was a positive subcutaneous culture (OR 10.4; 95% CI 3.5–30.4; *P*<0.001).

**Conclusion:**

Intraoperative subcutaneous cultures before skin closure may help early prediction of surgical site infection in open gynecological procedures.

## Introduction

Surgical site infection (SSI) is a major health issue around the world. It represents one of the most common causes of morbidity in the gynecological patient who encounters open surgery, occurring in approximately 2–5% of cases [1]. SSI rates according to traditional classification types stand at 2.1% for clean surgery, 3.3% for clean–contaminated, 6.4% for contaminated, and 7.1% for dirty or infected cases [2, 3].

The risk of SSI in the surgical patient is influenced by multiple factors that can be classified as intrinsic and extrinsic [4]. Local intrinsic factors include tissue injury produced during surgery, existence of nonviable or necrotic tissue, presence of foreign bodies, and skin microbiological colonization. Systemic intrinsic factors include circumstances involving tissue hypoxia, advanced age, blood transfusion, malnutrition, obesity, diabetes, other chronic diseases, smoking, steroids and other immunosuppressants [5]. As for the extrinsic factors, surgery lasting more than two hours, lack of antibiotic prophylaxis or intraoperative contamination which depends among other factors on the type of surgery (clean, clean-contaminated, contaminated and dirty) [6]. This classification is based on the estimated degree of bacterial contamination of the wound margins during surgery prior to closure; therefore, the presence of microorganisms in the surgical wound during surgery could determine an increased risk of SSI. There are studies that relate microbial colonization of the patient's skin prior to surgery to the risk of infection [2] but we did not find studies that investigate the relationship with the presence of microorganisms in the surgical incision, prior to closure.

In gynecology, there is an increasing interest in designing better surgical-specific bundles that help to reduce the rates of SSI [7, 8]. Identifying the factors related to SSI occurrence is essential to achieve this goal.

The aim of our study was to analyze the relationship between positive skin/subcutaneous cultures taken just before skin closure and SSI in women undergoing laparotomy for gynecological procedures.

## Material and methods

Hospital Universitario Quironsalud Madrid is a general university hospital with 291 inpatient beds, located in the west of the city of Madrid (Spain). All the media used in the study were provided by the hospital. The study was approved by Fundación Jiménez-Díaz Clinical Research Ethics Committee (reference number: 2017/24). Patients selected for the study were required to sign an informed consent. In the case of minor patients, the consent was signed by their parents or legal guardians.

We performed a prospective observational cohort study over a six-year period (January 2012—December 2017). All data were prospectively collected, with a follow up of patients in the first year after surgery.

Inclusion criteria: patients who underwent elective laparotomy for gynecological surgery at our hospital and signed informed consent. Surgical indications for laparotomy included: big pelvic, uterine or adnexal masses; severe endometriosis; early stage cervical cancer; advanced stage ovarian or endometrial cancer and intraperitoneal carcinomatosis of unknown origin.

Exclusion criteria: interventions in emergencies related to pelvic inflammatory disease, acute abdomen, ectopic pregnancy and bowel obstruction.

The preventive measures for SSI in gynecological patients undergoing laparotomy included: Perioperative normothermia to approximately 37° C, pre and post-operative glycemic control using blood glucose target levels < 200 mg/dL, preoperative antibiotic prophylaxis within one hour of surgical skin incision, using intravenous Cefazolin 2 g for patients < 120 kg and 3 g if >120 kg, and every four hours for lengthy procedures or excessive blood loss (>1500 ml); for patients with other high-risk factors, such as anticipated intestinal surgery, we used preoperative mechanical bowel preparation, without oral antibiotics; and intravenous Amoxicillin-Clavulanic acid 2 g, as antibiotic prophylaxis; for women with a history of allergic reaction to penicillin, metronidazol 500 mg plus gentamicin 5 mg/kg was used as an alternative, preoperative surgical site skin and vagina preparation and cleansing: first wipe using povidone-iodine soap and two additional wipes using 10% povidone-iodine scrub; maintain appropriate aseptic technique for scrubbed personnel, including surgeons, assistant, nursing staff and students and strict 24 hours dressing removal by house staff and/or nursing.

In January 2012, the collection of samples for microbiological analysis of subcutaneous tissue and skin before surgical wound closure was included in the elective laparotomy protocol.

The data collection protocol included: age, tobacco consumption, diabetes mellitus, previous chemotherapy, benign pathology or oncologic surgery, previous abdominal surgery, length of intervention (in minutes), type of incision, clean-contaminated surgery or contaminated surgery, intestinal resection, subcutaneous adipose tissue thickness (millimeters), subcutaneous or abdominal drainages, blood transfusion, length of hospitalization (days), results of microbiological cultures of skin and subcutaneous tissue intraoperative samples and presence of SSI, specifying type of infection (superficial incisional, deep incisional, and deep or organ-space infection) and etiology. Data were collected at the end of the surgical procedure except for the length of hospitalization, intraoperative culture results, SSI type and etiology that were collected throughout the patients' follow-up from the electronic medical record.

Clean-contaminated wounds are those involving a hollow muscular area without significant spillage of indigenous microbiota. Gastrointestinal tract incisions with a minimal number of microbes contaminating normally sterile tissue are considered clean-contaminated wounds. Contaminated wounds are those involving tissue with acute inflammation but no pus, or those with gross spillage from a hollow, muscular organ. Contaminated wounds also include those involving a major break in aseptic technique [9]. We based our criteria for defining and classifying surgical site infections on Centers for Disease Control and Prevention National Health Safety Network criteria [9]. SSIs were diagnosed within the first month after the surgical intervention. The primary surgeon involved in the procedure was in-charge of patients’ daily postoperative visits, including the diagnosis and surveillance of SSI. Once the patient was discharged, follow-up was carried out in the gynecological outpatient offices or in the emergency room.

### Microbiological assay

Subcutaneous samples were taken using a swab and rubbed throughout the wound just after closing the fascia and prior to skin closure. Skin samples were taken using the same method immediately after skin closure. The samples were then transferred to the laboratory and processed in blood agar, chocolate agar, MacConkey agar, CAN agar, and enrichment broth. A Sabouraud agar was added in order to rule out the presence of fungi. The plates were incubated at 35–37°C for 24–48 hours. The plates of blood agar, chocolate agar and CAN agar were incubated with 5–10% of CO_2_.

The culture broths were incubated for a minimum of four days. When turbidity was observed, a subculture to solid media was carried out.

Processing of samples from surgical wound infections: samples were aspirated and/or swabbed. These samples were transferred to the Microbiology laboratory immediately. The samples were processed in blood agar plates, chocolate agar, MacConkey agar, CAN agar and, enrichment broth. A Sabouraud agar plate in order to rule out the presence of fungi. The plates were incubated at 35–37°C for 24–48 hours. The plates of blood agar, chocolate agar and CAN agar were incubated with 5–10% of CO_2_. The culture broths were incubated for a minimum of four days. When turbidity was observed, a subculture to solid media was carried out. The growth of microorganisms essentially considered pathogens, such as *Staphylococcus aureus*, *β-hemolytic Streptococcus*, anaerobic bacteria, Enterobacteria and *Pseudomonas aeruginosa*, was always deemed significant. Isolation of coagulase-negative staphylococci or *Enterococcus spp*. had value in those samples in which these microorganisms were found in pure culture.

### Statistical analysis

Continuous variables are expressed as mean and standard deviation (SD), or as median and interquartile range (IQR) depending on their distribution. Categorical variables are expressed as numbers and percentages. For univariate analysis, t-test was performed for comparison of quantitative parametric variables and a Mann-Whitney-U test for the non-parametric ones. A chi-square analysis was used for qualitative variables. In univariate analyses, a *P* value of <0.1 was considered significant for inclusion in the multivariate model. Stepwise logistic regression with backward elimination was used to develop the multivariate models. Significance was based on alpha<0.05. Data analysis was performed with the SPSS software for Windows version 21 (IBM Corporation, Chicago, IL, USA).

## Results

We included 284 women who underwent laparotomy for gynecological surgery. Their ages ranged from 17 to 89 years. 179 (63%) of them were operated for oncologic pathology. The surgical procedure was “clean-contaminated” in all cases. All of them received antibiotic prophylaxis.

SSI occurred in 20 cases of 284 patients (7%). Eleven were superficial incisional infections (55%) and nine (45%) organ-space infections. SSI type and etiological profile are reported in Table 1.

**Table 1:** Surgical site infections: Type of infection and microorganism isolated (n = 20).

Type of infection and microorganism	n
Superficial incisional infection	
*E*. *coli*	2
*S*. *aureus*	1
*P*. *aeruginosa*	1
*S*. *lugdunensis*	2
*E*. *cloacae*	1
*E*. *faecalis*	1
*K*. *pneumoniae*	1
*P*. *aeruginosa* and *E*. *faecium*	1
Not performed	1
Organ- space infection	
Intra-abdominal infection	
*E*. *coli*	2
*E*. *coli*, *E*. *aerogenes*, *P*. *aeruginosa* and *E*. *avium*	1
Not performed	1
Intra-abdominal infection and bacteremia	
*E*. *coli* (blood); *E*. *coli* and *E*. *faecalis* (ascitic fluid)	1
*C*. *krusei* (blood); *P*. *aeruginosa*, *Bacteroides sp*. and *E*. *coli* (ascitic fluid)	1
Superficial incisional and intra-abdominal infection	
*E*. *coli* and *K*. *pneumoniae*	1
Superficial incisional and bacteremia	
*E*. *coli*	1
Bacteremia	
*P*. *aeruginosa*	1

At univariate analysis (Table 2), colon resection (OR = 3; 95% CI (1.2–7.6)), transfusion (OR = 3; 95% CI (1.2–7.5)), positive results of intraoperative skin cultures (OR = 4.1; CI 95% (1.3–12.3)), positive results of intraoperative subcutaneous cultures (OR = 10.4; CI 95% (3.5–30.4)) and length of surgery were found to be significantly associated with SSI occurrence. As regards length of surgery, we performed additional analyses comparing groups (less than 120 minutes, between 120 and 200 minutes and more than 200 minutes). We found significant differences in the comparison between surgeries lasting more than 200 minutes (11.7% with SSI) and those lasting less than 120 minutes (3.4% with SSI). OR = 3.7 (95% CI = 1.1–12.5); *P* = 0.025.

**Table 2:** Surgical site infection. Univariate analysis.

Characteristic	Surgical site infection		P^*^
	Yes	No	
	n = 20	n = 264	
Age (mean±SD)^b^	52.1±12.1	52.8±13.9	0.81
Current smoking^a^	4 (20%)	38 (14.4%)	0.51
Diabetes mellitus^a^	0	7 (2.7%)	1
Previous chemotherapy^a^	3 (15%)	68 (25.8%)	0.28
Oncologic surgery^a^	15 (75%)	164 (62.1%)	0.25
Colon resection^a^	10 (50%)	56 (21.2%)	0.01
Type of incision^a^			
Transverse skin/vertical subumbilical	1 (5%)	54 (20.5%)	0.22
Vertical paramedian	15 (75%)	173 (65.5%)	
Pfannenstiel	4 (20%)	37 (14%)	
Subcutaneous drainage^a^	4 (20%)	81 (30.7%)	0.31
Intra-abdominal drainage^a^	14 (70%)	126 (47.7%)	0.06
Transfusion^a^	9 (45%)	57 (21.6%)	0.03
Subcutaneous tissue thickness (mm) (median and IQR)^c^	30 (20–40)	25 (20–40)	0.4
Surgery duration (min) (median and IQR)^c^	182.5 (137.7–243.7)	145 (90–210)	0.047
Hospitalization days (median and IQR)^c^	5 (4–12)	5 (4–7)	0.16
Positive subcutaneous intraoperative culture^a^	7 (35%)	13 (4.9%)	<0.001
Positive skin intraoperative culture^a^	5 (25%)	20 (7.6%)	0.02

*P-values were calculated using chi-square test for qualitative variables^a^, *t*-test for quantitative parametric variables^b^ and Mann-Whitney-U test for the non-parametric ones^c^.

We found a statistically significant association between the positive results of intraoperative skin and subcutaneous cultures and the presence of SSI. SSI occurred in 35% (7/20) of patients with positive subcutaneous culture *versus* 4.9% (13/264) with normal microbiota or negative culture (*P*<0.001). Patients with positive skin culture had SSI in 20% (5/25) of cases *versus* 5.8% (15/259) with normal microbiota or negative culture (*P* = 0.02).

The results of intraoperative skin and subcutaneous tissue samples cultures are shown in Table 3. Skin cultures were negative in 41.5% and normal skin microbiota was isolated in 50.3% of patients. The microorganisms most frequently identified in the positive cultures were *E*. *coli* and *E*. *faecalis*. Subcutaneous tissue cultures were negative in 48.6% and normal skin microbiota was found in 44.3%. The microorganisms most frequently isolated were *K*. *pneumoniae*, *E*. *faecalis and E*. *coli*.

**Table 3:** Microorganisms isolated in skin and subcutaneous intraoperative cultures.

Skin	n = 284
*E*. *coli*	6 (2.1%)
*E*. *faecalis*	5 (1.8%)
*K*. *pneumoniae*	4 (1.4%)
*S*. *maltophilia*	2 (0.7%)
*E*. *cloacae*	2 (0.7%)
*E*. *coli and E*. *faecalis*	2 (0.7%)
*E*. *aerogenes*	1 (0.4%)
*P*. *aeruginosa*	1 (0.4%)
Normal skin microbiota	143 (50.3%)
Negative	118 (41.5%)
Subcutaneous tissue	n = 284
*K*. *pneumoniae*	5 (1.8%)
*E*. *faecalis*	4 (1.4%)
*E*. *coli*	4 (1.4%)
*C*. *albicans*	1 (0.4%)
*E*. *aerogenes*	1 (0.4%)
*P*. *aeruginosa*	1 (0.4%)
*K*. *pneumoniae* and *P*. *aeruginosa*	1 (0.4%)
*E*. *coli* and *E*. *faecalis*	1 (0.4%)
*P*. *aeruginosa*, *E*. *coli* and *E*. *faecalis*	1 (0.4%)
*P*. *aeruginosa* and *E*. *faecium*	1 (0.4%)
Normal skin microbiota	126 (44.3%)
Negative	138 (48.6%)

Multivariate analysis (Table 4) showed an independent and statistically significant association of SSI with positive intraoperative subcutaneous tissue culture (OR 10.4; 95% CI = 3.5–30.5; *P*<0.001).

**Table 4:** Surgical site infection. Multivariate analysis.

	Adjusted OR (95% CI)	*P*
Positive subcutaneous tissue culture	10.4 (3.5–30.5)	<0.001
Colon resection	2.3 (0.8–6.5)	0.11
Transfusion	1.5 (0.5–4.7)	0.44
Surgery duration	1 (0.99–1.01)	0.5
Intra-abdominal drainage	1.4 (0.4–5.2)	0.66
Positive skin culture	1.3 (0.3–5.7)	0.68

## Discussion

This study shows a significant association between a positive culture of subcutaneous tissue specimen obtained just before skin closure, and the development of SSI, in gynecological patients undergoing laparotomy. The global frequency of SSI in our sample was 7% while the rate of SSI in patients with positive subcutaneous intraoperative culture was 35%.

In the univariate analysis, we identified risk factors for SSIs previously described [4]. These factors included need for transfusions [10–12], colon resection and duration of surgery [10]. The CDC [9] define a class III/contaminated surgery as an operation with major breaks in sterile technique, including gross spillage from the gastrointestinal tract. The National Nosocomial Infections System [13] considers contaminated surgery as one of the indicators to determine an infection risk index. In our study, all surgical procedures were classified as clean-contaminated but we found an association between increased risk of SSI and colon resection at univariate analysis. Other indicators include the American Society of Anesthesiologists’ physical status (ASA) greater than 2, and the procedure specific operative time greater than the 75^th^ percentile (for example: two hours for hysterectomy). We found a higher rate of postoperative SSI related to length of surgery. Interventions lasting more than 200 minutes had three times more frequency of infection than those lasting less than 120 minutes. In fact, long operative time is a well-known risk factor associated with SSIs, as confirmed in previous studies [10–12]. This risk is probably not only related to surgical complexity, but also to other factors such as increased operating room traffic [10–14], increased risk of surgeon’s glove breakage, and other disruptive events affecting appropriate aseptic technique [15].

Perioperative blood transfusion is frequently included as another risk factor for SSIs [10, 11]. We also found a higher risk of postoperative infection complications in patients who received blood products. The rationale behind the higher risk of SSI in anemic patients is probably related to lower tissue oxygen tension because of low levels of hemoglobin carrying capacity [16, 17]. For lengthy procedures or cases with excessive blood loss (greater than 1500 ml), additional intraoperative re-dosing of prophylactic antibiotics is justified [12].

Peritoneal drainage is another controversial measure traditionally used by general and pelvic surgeons to prevent intra-abdominal accumulation of fluid, to evacuate blood or as a sentinel alert in surveillance for early discovery of anastomotic leakage or bleeding. However, there is no scientific evidence that drainage gives better outcomes after abdominopelvic surgery [15–18]. The Enhanced Recovery After Surgery (ERAS®) Society [19] does not recommend routine peritoneal drainage for patients undergoing gynecologic/oncology surgery, including lymphadenectomy or bowel surgery. In our study we did not find a significant association between the use of drains and SSI.

Following wound closure, skin epithelization occurs in 24 hours. From then on, probability of outside bacterial contamination of the surgical site is extremely low. Based on this fact, and on a 2015 Cochrane review [20], we removed surgical dressings within the first 48 hours. We believe that surgical site bacterial contamination probably occurs during surgery and not during postoperative wound management. Therefore, efforts to prevent SSIs should focus on pre- and intraoperative measures.

Hospitals that treat complex patients (elderly, obese, diabetic, malnourished, oncologic or immunocompromised), who in addition undergo long operations or require blood transfusions, would probably need new elements in the SSI prevention bundles already described in the gynecological literature [7, 8]. The inclusion of subcutaneous cultures at skin closure may be a new component of these bundles. It is likely that these patients may have a higher predisposition for bacterial colonization and, therefore, have an increased risk for SSI when undergoing open surgery. The positive result of subcutaneous culture can be available within 24–48 hours after surgery and may help predict this surgical complication, in this group of high-risk patients. Another potential use of subcutaneous cultures to predict surgical site infection is vulvar [21] and inguinal [22] surgery. These are also high-risk procedures for postoperative infectious complications and further studies conducted on patients undergoing this type of surgery could clarify this issue.

In this study, we found that a positive subcutaneous culture was an independent variable associated with SSI. Although there were more patients with positive skin intraoperative culture than positive subcutaneous culture, this variable (positive skin culture) was not independently associated with SSI. Adipose tissue can be a good culture media for bacterial overgrowth, specifically when dealing with an immunocompromised host.

On the other hand, minimally invasive surgery has emerged as a new approach to the surgical management. One of the major advantages of laparoscopic or robotic surgery is low SSI rates in comparison with laparotomy [23]. However, there are still many indications for open surgery in the gynecological field, especially in oncology patients.

A potential weakness of the study is the lack of information about preoperative nutritional status, body mass index, ASA status or intraoperative glycemic control. Currently, CDC guidelines [9] for SSI prevention recommend performing intraoperative skin preparation with an alcohol-based antiseptic agent. During the study period, the preoperative surgical site skin and vagina preparation was performed using povidone-iodine. This may affect the external validity of our results.

As far as we know, this is the first study investigating the relationship between the results of intraoperative cultures and the development of SSI. According to our results, the presence of positive intraoperative subcutaneous culture could be useful to predict an increased risk of SSI in patients undergoing gynecological laparotomy.

## Supporting information

S1 File SSI dataset. (ZIP)
